# 

**Published:** 1878-11

**Authors:** 


*CONTENTS.*

| | Page |
|---|---|
| Sweet Forbearance . . . | 629 |
| Spare the Birds . . . . . | 630 |
| Letter from a Sick Room . | 632 |
| The Joking Clergyman . . | 633 |
| The Sovereigns of England | 636 |
| Natural Death . . . . . | 637 |
| Our Changing Climate . . | 639 |
| Mental Epidemics . . . . | 640 |
| Dangerous Newspaper Reading | 646 |
| Drowning at Sea . . . . | 650 |
| Bread . . . . . . . . . . | 652 |
| Poisons . . . . . . . . . | 652 |
| Cure of Bites and Stings . . | 652 |
| Debt and Death . . . . . | 652 |
| Money and Mind . . . . . | 654 |
| Characteristics of Doctors . | 654 |
| Disparity of Age in Marriage . . . . . . . | 654 |

